# Filamentous Fungal Human Pathogens from Food Emphasising *Aspergillus*, *Fusarium* and *Mucor*

**DOI:** 10.3390/microorganisms5030044

**Published:** 2017-08-02

**Authors:** R. Russell M. Paterson, Nelson Lima

**Affiliations:** CEB—Centre of Biological Engineering, University of Minho, Campus de Gualtar, 4700-057 Braga, Portugal; nelson@ie.uminho.pt

**Keywords:** cereals, database, dairy, food, mycosis, nuts, plant pathology, *Aspergillus*, *Fusarium*, *Mucor*

## Abstract

Disease caused by filamentous fungal human pathogens (FFHP) is increasing. These organisms cause severe mycoses in immunosuppressed individuals, such as those: (a) with AIDS; (b) having undergone transplantation; and/or (c) undergoing chemotherapy. Immunocompetent people can become infected. Some FFHP are isolated from foods which may be fomites. However, the information concerning particular species on specific food is large, dispersed and difficult to obtain. Reports of filamentous fungi from food/crops and causing human disease are frequently only available in the literature of food mycology/plant pathology and medical mycology, respectively: it is seldom cross-referenced. *Aspergillus* contains some species with strains that are the most dangerous FFHP, with *Aspergillus fumigatus* causing the most serious diseases. *Fusarium* and *Mucor* also contain species of high importance and approximately 15 other genera are involved. A checklist and database of FFHP species isolated from food is presented herein with emphasis on *Aspergillus*, *Fusarium* and *Mucor* in summary tables to increase awareness of the connection between food and FFHP. Metadata on all FFHP is provided in a large supplementary table for updating and revision when necessary. Previous names of fungi have been revised to reflect current valid usage whenever appropriate. The information will form a foundation for future research and taxonomic revisions in the field. The paper will be highly useful for medical practitioners, food mycologists, fungal taxonomists, patients, regulators and food producers interested in reducing infectious diseases and producing high quality food.

## 1. Introduction

Diseases caused by filamentous fungal human pathogens (FFHP) have increased considerably [1], although they remain underestimated [2]. Immunosuppressed people are susceptible, such as individuals with AIDS [3] and those undergoing transplantation and chemotherapy, although immunocompetent individuals can succumb [4]. The presence of these fungi in/on foods represents a threat to patients as fomites [4,5,6,7,8,9].

For example, Liu, 2011 [1] contains chapters on ca. 70 genera of FFHP, and 15 (ca. 21%) of these are recorded from food frequently [4]. FFHP cause localized infection of the lungs, sinuses, and other sites in non-immunocompromised people. Human diseases result from inhalation of conidia, which can exacerbate allergic symptoms in atopic and non-atopic individuals [10,11,12]. Invasive pulmonary disease usually only occurs in immunocompromised patients with inhalation being the primary source of infection. Gastrointestinal routes are sources of infection for fungi and many precautions are already made enabling patients to avoid eating suspect foods [4,5,6,7,8,9].

Strains of particular *Aspergillus* species are the biggest threat [1,4] where *Aspergillus fumigatus* is the most important FFHP, involving 85–90% of invasive aspergillosis. This is one of the leading causes of mortality in patients: (a) with haematological malignancies; and (b) receiving intensive chemotherapy and hematopoietic stem cell transplantations [7,8]. *Aspergillus flavus* is the second most important, followed by *A. niger*, *A. terreus* and *A. nidulans*.

Some *Mucor* species contain strains which are significant infective agents of immunocompromised patients [13,14], although it is probable that high numbers of cases are unrecorded from difficulties with identification of taxa [9,10], which would be assisted by knowledge of those recorded previously. Interestingly, potentially human infective strains of *Mucor circinelloides* have been isolated recently from yogurts, which were the presumed causal organism for illness in >200 consumers [15]. The increase of immunocompromised cohorts has seen a higher rate of mucormycosis and ca. 15% of patients suffering severe neutropenia developed mucormycosis, with mortality from 68% to 100%. The main infection sites are lungs, sinuses, soft tissues, skin, and bloodstream. Gastrointestinal (GI) mucormycosis case reports have been published where transplant recipients are susceptible, especially following ingestion of the causative fungal species, e.g., a case was reported from a patient who ingested naturopathic medicine contaminated with *Mucor* [16]. The mortality rate of GI mucormycosis can be 85%, and the infection is often disseminated, resulting in even higher rates [15].

Species of *Fusarium* have emerged as an increasingly important causal agent of opportunistic infections in humans [17]. The epidemiological distribution of fusariosis is controversial and a large number of institutions worldwide have only occasionally, or never, documented invasive *Fusarium* infection, although it is well recorded in the USA, Brazil, France and Italy. It is likely that *Fusarium* colonises patients prior to hospital admission and subsequent immunosuppression and neutropenia results in infections. Food may represent a source of infection.

The other pathogenic genera involved from food include: *Lichtheimia* [18,19]; *Curvularia* [20,21]; *Phoma* [22,23]; *Trichoderma* [24,25]; *Alternaria* [26,27]; *Acremonium* [28,29]; *Paecilomyces* [30,31]; *Penicillium* [32]; *Achaetomium*/*Amesia*/*Botryotrichum*/*Chaetomium*/*Dichotomopilus* [33,34,35]; *Microascus*/*Scopulariopsis* [36,37]; and *Wallemia* [38,39] (Table S1), and prior knowledge of pathogenic species from foods will assist in future investigations.

The information on filamentous fungi (FF) from food/crops and causing human disease is available often only in the literature of food mycology/plant pathology and medical mycology, respectively, without researchers being aware sufficiently of each alternative source of data. Medical researchers may isolate fungi from food de novo (e.g., [5,6,7,8,9]), perhaps unnecessarily in some cases, as the information already exists, albeit in a dispersed manner. Furthermore, food mycologists underestimate the significance of FFHP from food in their quest for high quality food, although species of *Aspergillus*, *Penicillium* and *Fusarium* are studied extensively often from the point of view of: (a) mycotoxin production [4,40]; (b) deterioration of organoleptic properties; or (c) plant pathology. This extensive source of information has been underutilized in medical mycology because it is presented in the food or plant pathology literature. To address this situation, Paterson and Lima [4] discussed FF isolated from food that also cause human disease; however, the data are not in a readily accessible form for all species and foods. Conversely, the reports of FFHP are from medical mycology, and not food mycology or plant pathology. The previous reports in the literature occasionally use fungal names that have been revised leading to confusion and difficulty in tracking the correct species involved in diseases.

This present report combines sources of information as a meta-database, whilst indicating which of the contaminated foods may be safer from containing fewer of the more dangerous FFHP. The database can be continually developed and revised. This list was compiled predominantly from information in [4] supplemented with that in [1,41,42]. Importantly, the names of the fungi reported previously in the literature have been updated to modern valid concepts by employing searches in Mycobank (http://www.mycobank.org/) and Index Fungorum (http://www.indexfungorum.org/names/names.asp), providing insights into the phylogeny of the taxonomic groups. A summary table is provided for *Aspergillus*, *Fusarium* and *Mucor* in the text (Table 1), while the complete database of all fungi is in Supplementary Materials (Table S1). These will be employed to increase awareness of the issue and assist medical practitioners, patients, patient assistants, regulators and food producers interested in reducing infectious diseases and producing high quality food.

## 2. Results and Discussion

Potentially pathogenic aspergilli, fusaria and *Mucor* spp. have been isolated from a wide range of foods ranging from baked goods to vegetables (Table 1 and Table S1). The data do not indicate the: (a) frequency of isolation of a species; nor (b) concentrations of fungi from a particular food. Additional information can be obtained from [1,4,41,42], but providing this is beyond the scope of the present paper.

### 2.1. Aspergilli

Aspergilli were reported in a total of ca. 90 foods (Table S1) which is higher than that for *Fusarium*, or *Mucor*. Seven species of aspergilli were recorded. *A. flavus* was listed the most from foods which may reflect its significance as the producer of the most important mycotoxins, the aflatoxins, in that the identification of this fungus would take precedence over others within food mycology. *A. fumigatus* was the second most frequent species listed from the foods and is of great importance as a human pathogen. *Aspergillus niger* was frequently found in foods in which aspergilli were detected, and is a producer of the important mycotoxins ochratoxin A and fumonisin B2.

*A. fumigatus* was reported frequently from cereals such as barley, rice and wheat and other individual foods. The fungus was common from nuts, being recorded from copra, hazelnuts, peanuts, and walnuts. *A. flavus* was again listed more frequently and was only not recorded from cashews and candle nuts (Table S1). *A. fumigatus* was reported from melon seeds and oilseeds, and *A. flavus* was from amaranth, oil, rape, sesame, and sunflower seeds. Bread was a source of *A. flavus*: *A. fumigatus* was isolated from baldi bread (Table S1). It is likely that freshly baked bread would be free of fungal contamination and not present a health risk. *A. flavus* was listed frequently from beans (Table S1) which could be a serious source of infection. Cocoa was reported as a source of *A. fumigatus*, whereas *A. flavus* and *A. niger* were recorded from coffee. *A. fumigatus* was found from low fat buffalo cheese and processed cheese: these may represent particular risks. *A. flavus* was reported from milk and cheese which may be worthy of further research. The foods mentioned above are likely sources of infection with these most dangerous FF.

Remarkably, *A. fumigatus* was unrecorded from fruit, except pickled mangoes, and fruit may be useful for immunocompromised individuals. *A. flavus* was common and found from dried fruit, citrus, litchis, pineapples, pomegranates and tomatoes, some of which are tropical. The fungus was seldom reported from more common fruits such as apples, bananas, grapes, pears and oranges (Table S1), some of which are from temperate climate crops. This difference might be explained by the species being associated with warmer climates. In addition, *A. fumigatus* is a thermophilic species and may not grow optimally in temperate climates. A recommendation is that fruits from temperate climates could be safer in a general sense, although more work is required.

Few herbs, spices and truffles were reported to contain *A. fumigatus*, which is noteworthy, although the general category of “spices” was contaminated. *A. flavus* is more common, with contamination of peppers being important. *A. fumigatus* was found from dried fish, stored eggs, and cured and processed meat (Table S1), and *A. flavus* had a different profile within this group of foods. These foods may pose particular risks. *A. fumigatus* was not reported from baked goods (except baldi bread which is a specialized commodity), beans (apart from soybeans), beverages (apart from cocoa), fruit (apart from pickled mangoes), and herbs (Table S1), and these may be safe from this perspective.

The maximum number of *Aspergillus* species detected in a particular commodity was three for corn/maize (including a snack) (*A. flavus* was one of the fungi detected), rice, hazelnuts, peanuts, and walnuts. These foods present a considerable risk. *A. fumigatus* and *A. flavus* were reported in soybeans, barley, rice, wheat, “spices”, copra, hazelnuts, peanuts, walnuts, and oilseeds, which consequently represent high risk foods.

### 2.2. Fusaria

Fusaria were reported from a wide range of food [17] (Table 1), particularly from fruit, beans, vegetables, and cereals, but were not listed in baked goods (Table S1). Only one case of the genus being isolated from dairy (*F. moniliforme* [current valid name is *F. verticillioides*] from cheese) and beverages (*F. oxysporum* from cacao beans and leaves, which together are considered as one substrate) was reported. Numerous species are recoded from herbs/spices/truffles: *F. oxysporum* was reported from truffles. Hence, these might be an important source of infections by this genus. Nuts are obvious fomites and species were reported frequently from seeds, with an overall profile similar to nuts. Fusaria were common throughout the vegetables, indicating they may be serious sources of infection.

*F. oxysporum* (Table S1) was most frequently isolated from foods of the fusaria recorded, which may relate to the involvement of this fungus in wilts of crops where they would be reported frequently in plant pathology related papers. *Fusarium verticillioides* was listed frequently (Table S1) and fungus is a well-known fumonisin mycotoxin producer, perhaps explaining why many foods are reported as contaminated by this fungus, because it is particularly important. Fumonisin contamination of corn and other cereals is primarily from *F. verticillioides*, which was typically reported as *F. moniliforme* until ca. 10 years ago. The trichothecene producing fungus *Fusarium graminearum* was only recorded from 0.8% of the foods, and may be rare in other foods apart from corn/maize. *Fusarium solani* was reported frequently which causes foot and root rot of diverse hosts and may explain its frequency of detection. Many of the foods had numerous species of *Fusarium* reported from them, including: (a) corn/maize, sorghum, bananas and peanuts (seven); (b) soybeans (six); and (c) coriander (four) (Table S1).

### 2.3. Mucor

Twenty-seven different foods were reported as contaminated with *Mucor* species which is considerably fewer than for fusaria and aspergilli. *Mucor* contains important pathogens and was associated particularly with dairy products (e.g., cheese, yoghurt [15] and soybeans (Table S1)). Four species were described from soybeans and cheese which represents the highest number of different *Mucor* species reported for single foods. Three species/taxa were recorded form yogurt. Hence, these foods may represent a high threat for *Mucor* diseases. *Mucor* spp. were common in cereals and grains, but were not recorded from rice, expect for paddy rice, and they were unrecorded from wheat, except for a single fast food. *M. racemosus* was listed the most from food at ca. 30%.

### 2.4. General Discussion

In general, the greatest sources of potential infection are nuts and cereals (Table S1) and these are well known to harbour biodeteriorating fungi. Foods grown close to the soil appear as more highly contaminated (e.g., vegetables, peanuts, and cereals) and soil is known as a frequent source of pathogenic FF [43]. The contamination of fruit may relate to the plant pathogenic activities of some human disease fungi, especially the fusaria. Finally, baked goods are almost free of the most infective fungi and appear to be safe, although the less important FFHP may be present (Table S1).

Soy products, butter, margarine, dried milk, condensed milk, jams, jellies, eggs, and salmon, amongst others, are examples of what may be safe products when Table 2 and Table S1 are considered. These have none of the three most important fungi described herein and only few of the lesser pathogens. However, further testing is required to establish their safety more clearly.

The foods from which aspergilli, fusaria and *Mucor* were not detected can be determined from Table S1 and Table 2: these may be considered as “low risk” under this present assessment. Table 2 is compiled from foods from which at least one pathogenic fungi was reported. Information on foods from which fungi were not isolated when tested would not be published in general. The current authors recommend these results are published in the future for the advancement of producing safe food. The foods from which aspergilli, fusaria and *Mucor* were not reported represent those foods from which at least one other FFHP was listed (Table S1), and hence cannot be considered as free from FFHP.

Bouakline et al. [5] considered that *Aspergillus* in food is an indirect source of airway or digestive tract colonisation of particular concern to haematology units. They provided a list of 36 foods from which fungi were isolated taken from ward kitchens, a central kitchen and a hospital pharmacy and focused on the aspergilli, where some were identified to species and *A. fumigatus* was prevalent. FF were far more frequent than yeasts. The others were identified as members of the Mucorales, *Trichoderma* and *Chaetomium*: pepper and tea were contaminated highly with aspergilli and members of the Mucorales.

In general, candidates undergoing conditioning therapy and recipients of haematopoietic cell transplantation (HCT) should avoid foods that increase the risk of exposure to fungi [6]: a table of foods that HCT recipients may eat and should avoid is provided. The foods groups include dairy, meat, meat substitutes, fruits, nuts, starters, soups, vegetables, bread, grains, cereals, beverages, desserts and others. Much of the information in [6] is devoted to yeasts and the FF diseases mentioned only include aspergillosis and fusariosis. Zygomycetes and *Scedosporium prolificans* are discussed indirectly, but which foods were associated with particular fungi was not indicated.

Sipsas and Kontoyiannis [7] describe the risk of particular lifestyles on invasive fungal (yeast and FF) infections and mention diet briefly. Brenier-Pinchart et al. [8] established a protocol for food management for allogeneic stem-cell transplant recipients in a protected ward in relation to FF. They confirm that other FF infections were rising apart from the known increase from *A. fumigatus* and confirm that the gastrointestinal tract might be a portal of entry from contaminated food ingestion or food intentionally containing fungi (e.g., blue cheese). Pepper and teas bags were prohibited and foods containing fungi as part of the food production process were recommended as to be avoided. FF were isolated every year from 1992 to 2002 in their analysis of protected food for patients.

Opportunistic fungal infections remain common consequences of cancer treatment [9], when the patients are often at home between treatments with uncontrolled access to foods, similar to people in the general population. *Alternaria*, *Aspergillus*, *Cladosporium*, *Mucor* and *Penicillium* spp. were reported from fresh fruits, mouldy cheese and smoked foods: consumption should be restricted during profound neutropenia. Eating the following requires to be avoided: (a) thick skinned fruit such as bananas, grapefruits and oranges (as the skin could contaminate the flesh); (b) other fruits such as apples and strawberries during neutropenia periods; and (c) ethnic spices and nuts including black and white pepper, Brazil nuts, almonds, cashews, chestnuts, hazelnuts, pistachios and walnuts. A list of recommendations of food to eat and not eat is provided, although yeast and FF are not distinguished and the foodstuffs are considered from the point of view of fungi in general, rather than specific taxa.

The data presented in Table S1 are consistent with the list of recommended and not recommended foods in [6]. For example, fruit and vegetables may be appropriate for HCT recipients if they are suitably washed and peeled, even if they were highly contaminated before preparation.

Bouakline et al. [5] mention that, for example, cereals, melon, apple juice and chocolate were not contaminated by fungi. However, FF have been isolated from these commodities (Table S1) and cereals were particularly frequently reported, as would be expected. Indeed, particular care is required with cereals. A wider range of fungi has been isolated (Table S1) from at least some of the commodities listed in [5]; hence, the threat from FF is greater than they indicated. The situation is different in [8] because the food they tested was highly controlled in an attempt to limit the fungal load. The food recommendations in [9] for patients with haematological cancers concur with the findings of the current paper, although more emphasis on avoiding untreated cereals would have been advantageous.

Cooking will destroy the problematic fungi in unprocessed food such as fresh vegetables, thus infections may occur before cooking and in the field. However, fresh vegetables are consumed frequently. It is important to point out that individual foods could cause particular problems despite not being considered a risk. For example, although dairy products appear safe, recent indication of yogurt as a source of infection by *Mucor* (Table S1 [15]) needs acknowledgment. Certain foods will be surveyed more often than others and it is likely that cereals and nuts are particularly highly sampled which will tend to bias results. In general, there is a lack of awareness that ingestion of contaminated food is a source of infection and that so many fungi may be involved. Propagules may enter the atmosphere from contaminated food for example in stored food in warehouses. Agricultural workers, or those working in fresh product markets, may be exposed in this manner, as could those preparing food for eating and cooking, although the risk may be considered as small on most occasions. If a person is exposed to large amounts of inocula over a long period of time, then even immunocompetent individuals may succumb (cf. farmer’s lung diseases). These infections are not from eating food per se.

Paterson and Lima [44] considered the effect of global warming on the presence of fungi on food and considered that thermotolerant and thermophilic fungi may dominate by succession over mesophilic fungi. *A. fumigatus* was considered the greatest risk from this perspective. Hence, diseases from this fungus may be more prevalent in the future. *A. flavus* may also present a greater future risk, especially in temperate countries in the near future.

It cannot be assumed that the identifications of the FF from food or from patients are correct in all cases. The identification of many FF is notoriously difficult [1,4,45] and there are misidentifications in the literature. The fungi are often variable in culture and identifications using morphology is often based on minute difference in the structure of conidiophores for example, which are difficult to distinguish even for experts. The tables presented herein act as a foundation from which further work can be undertaken. The present paper provides more detailed information of the taxa of most concern and on which foods they may be present from data that already exists in the literature. It is important that these data are referred to in addition to fungal isolations and identifications undertaken de novo.

## 3. Conclusions

The prevalence of FFHP from food makes it a highly probable source of infection for mycoses and the species of FF that cause the most serious infections are from a wide variety of foods. Not all foods were equally reported to have fungi associated with them and this may indicate which foods are safe and unsafe. Some foods did not have the three main fungi discussed herein reported from them. More work is required to determine definitely the safety of foods from the perspective of FF. This current paper provides a useful checklist to those involved in protecting patients from FF diseases.

## Figures and Tables

**Table 1:** Detection of human pathogenic fungi from various food samples.

Food Category	Fungi								
	*Af*	*Afu*	AO	*Fo*	*Fv*	FO	*Mc*	*Mh*	*M*O
**Baked Goods**	+	+							+
**Beans**	+	+	+	+		+	+	+	+
**Beverages/Chocolate**	+	+	+	+				+	+
**Cereals**									
Barley	+	+		+		+	+		+
Corn/Maize	+	+	+	+	+	+	+	+	
Millet	+					+			
Oats	+								
Rice	+	+	+	+	+	+		+	+
Sorghum	+			+		+			
Wheat	+	+			+	+		+	
**Dairy/Margarine**	+	+	+			+	+	+	+
**Fruit**	+	+	+	+		+	+	+	+
**Herbs/Spices/Fungi**	+	+	+	+		+			+
**Meat/Eggs/Fish**	+	+	+			+	+		+
**Nuts**	+	+	+	+		+	+	+	+
**Seeds**	+	+	+	+		+			
**Vegetables**	+	+	+	+		+	+	+	+

N.B. Other foods were tested and were negative for these fungi and this full list is provided in Table S1. *Af* = *A. flavus*; *Afu* = *A. fumigatus*; AO = other *Aspergillus* species; *Fo* = *F. oxysporum*; *Fv* = *F. verticillioides*; FO = other *Fusarium* species; *Mc* = *M. circinelloides*; *Mh =* *M. hiemalis*; *M*O = other *Mucor* species.

**Table 2:** Foods from which aspergilli, fusaria and *Mucor*, the most important taxa in this context, were not recorded. However, these foods have at least one other less serious filamentous fungal pathogen recorded from them (Table S1). There may be occasions where foods have been tested for filamentous fungi and none have been isolated. These negative results are unavailable to the scientific community, as they would not be published in general.

Food Category	Specific Foods
Baked Goods	Ginger bread, Marzipan cake
Beans	Soy products (Mejia/Noodles/Nuruk)
Beverages/Chocolate	Beverages (pasteurised), Tea black/green
Cereals	Buckwheat, Wheat (fermented)
Dairy/Margarine	Butter, Margarine, Dried milk (non-fat), Condensed milk
Fruit	Stored apples, Apple peel (Lenticel), Banana (Latundan), Dates, Jams, Jellies, Pears, Prunes, Dried sultanas
Herbs/Spices/Truffles	Bishop’s weed (herbal medicine), Caraway, Cinnamon, Cumin, Fennel, Ginger, Hops, White pepper
Meat/Eggs/Fish	Eggs, Fish meal, Ham, Meat (cold/frozen), Dried cured meat, Salmon, Salami, Suet
Nuts/Seeds	Cashew nuts, Candle nuts, Cucumber seeds, Legume seeds, Pea seeds, Pine seeds, Pumpkin seeds, Safflower seeds
Vegetables	Cassava (rotting), Cauliflowers, Linseed, Maple syrup, Oils, Okra, Olive paste, Peas dried, Radish, Sugarcane

## Supplementary Materials

The following are available online at www.mdpi.com/2076-2607/5/3/44/s1, Table S1: Human pathogenic filamentous fungi isolated recorded from food. Names that are currently invalid but found in the literature are indicated by square brackets.
