# Supplementary material for: Filamentous Fungal Human Pathogens from Food Emphasising Aspergillus, Fusarium and Mucor
Source: Microorganisms. 2017 Aug 2;5(3):44. doi: 10.3390/microorganisms5030044 (PMC5620635; doi:10.3390/microorganisms5030044)
Supplement: Supplementary file 1 [file microorganisms-05-00044-s001.pdf]

Table S1. Human pathogenic filamentous fungi recorded from food. Names that are currently invalid but found in the literature are placed in square brackets for reference.

Food Category	Fungi
**Baked Goods**	
Bread	*Aspergillus flavus, Penicillium roqueforti, Wallemia sebi*
Bread (baldi)	*Aspergillus fumigatus*
Cheesecake	*Mucor racemosus*
Ginger bread	*Wallemia sebi*
Marzipan cake	*Wallemia sebi*
**Beans**	
Beans (unspecified)	*Alternaria alternata, Dichotomopilus funicola [Chaetomium funicola], Fusarium incarnatum [F. semitectum], Fusarium solani, Chaetomium globosum [C. cochliodes], C. indicum*
Beans (black)	*Aspergillus flavus, Dichotomopilus funicola, Chaetomium brasiliense, C. globosum*
Beans (mung)	*Aspergillus flavus, Dichotomopilus funicola, Chaetomium brasiliense, C. globosum, Fusarium chlamydosporum, F. verticillioides [F. moniliforme], F. proliferatum, Mucor circinelloides, Scopulariopsis brevicaulis [Microascus brevicaulis], Wallemia sebi*
Beans (velvet)	*Aspergillus flavus*
Beans (talo)	*Aspergillus flavus*
Cowpeas	*Aspergillus flavus, A. ochraceus, Fusarium equiseti, F. oxysporum, Trichoderma harzianum*
Soy products (Mejia/Noodles/Nuruk)	*Lichtheimia corymbifera, L. hyalospora, L. ornata, Scopulariopsis brevicaulis, Wallemia sebi*
Soybeans	*Alternaria alternata, Aspergillus flavus, A. fumigatus, Chaetomium brasiliense, C. globosum, Dichotomopilus funicola, Fusarium equiseti, F. incarnatum, F. oxysporum, F. solani, F. verticillioides, Mucor circinelloides, M. hiemalis, M. plumbeus, M. racemosus, Scopulariopsis brevicaulis, Wallemia sebi*
**Beverages/chocolate**	
Beverages (pasteurised)	*Paecilomyces variotii*
Cacao beans and leaves	*Fusarium oxysporum, Scopulariopsis asperula [Microascus niger]*
Cacao beans fermenting	*Mucor racemosus*
Cocoa	*Aspergillus fumigatus*
Chocolate	*Mucor hiemalis*
Coffee	*Microascus cinereus*
Coffee beans (green)	*Aspergillus flavus, A. niger*
Coffee: monsoon	*Aspergillus niger*
Tea black/green	*Chaetomium globosum*
**Cereals**	
Cereals/Grains	*Acremonium strictum, Aspergillus clavatus, Fusarium equiseti, Microascus cirrosus, M. manginii* (anamorph *Scopulariopsis candida*), *M. trigonosporus, Mucor plumbeus, Paecilomyces variotii, Trichoderma harzianum, Wallemia sebi*
Barley	*Alternaria alternata, Al. infectoria, Aspergillus flavus, A. fumigatus, Dichotomopilus funicola, Chaetomium brasiliense, C. globosum, Curvularia lunata, Fusarium equiseti, F. oxysporum, Scopulariopsis brevicaulis, Mucor circinelloides, M. racemosus*
Buckwheat	*Microascus manginii*

7 Corn/Maize	*Acremonium strictum, Aspergillus flavus, A. niger, Chaetomium brasiliense, C. globosum, Curvularia lunata, Dichotomopilus funicola, Fusarium equiseti, F. oxysporum, F. graminearum, F. incarnatum, F.solani, F. proliferatum, F. verticillioides, Scopulariopsis brevicaulis, Microascus cinereus, M. cirrosus, M. manginii, Mucor circinelloides, M. hiemalis Trichoderma harzianum, Wallemia sebi*
Corn snacks	*Aspergillus fumigatus*
Millet	*Aspergillus flavus, Chaetomium globosum, Fusarium chlamydosporum*
Oats	*Aspergillus flavus, Scopulariopsis brevicaulis, Microascus cinereus*
Pasta	*Aspergillus flavus*
Rice	*Alternaria alternata, Amesia atrobrunnea [Chaetomium atrobrunneum], Aspergillus flavus, A. fumigatus, A. niger, Botryotrichum murorum [Chaetomium murorum], Chaetomium brasiliense, C. globosum, Curvularia lunata, Dichotomopilus funicola, Fusarium equiseti, F. oxysporum, F. verticillioides, Scopulariopsis brevicaulis, Wallemia sebi*
Rice bran	*Aspergillus flavus*
Rice milled	*Aspergillus flavus, Microascus manginii, M. trigonosporus, Scopulariopsis brevicaulis*
Rice (parboiled)	*Aspergillus fumigatus*
Rice (paddy)	*Aspergillus flavus, Curvularia lunata, C. pallescens, Fusarium incarnatum, F. proliferatum, F. solani, Mucor hiemalis, M. racemosus*
Rice (black)	*Fusarium incarnatum, Mucor plumbeus*
Rye	*Alternaria infectoria, Fusarium equiseti, Paecilomyces variotii*
Sorghum	*Acremonium strictum, Alternaria alternata, Aspergillus flavus, Chaetomium brasiliense, C. globosum, C. pallescens, Curvularia lunata, Dichotomopilus funicola, Fusarium chlamydosporum, F. incarnatum, F. oxysporum, F. proliferatum, F. solani, F. verticillioides, Microascus trigonosporus, Phoma sorghina, Trichoderma harzianum*
Wheat	*Acremonium egyptiacum, Alternaria alternata, Aspergillus flavus, A. fumigatus, Chaetomium globosum, Curvularia lunata, Fusarium equiseti, F. verticillioides, Microascus trigonosporus, Penicillium citrinum, Scopulariopsis brevicaulis, Wallemia sebi*
Wheat (fermented)	*Scopulariopsis asperula*
Wheat-based fast food	*Mucor hiemalis*
Wheat flour	*Aspergillus flavus, Microascus cinereus, Scopulariopsis asperula*
**Dairy/Margarine**	
Butter	*Scopulariopsis brevicaulis*
Cheese	*Aspergillus flavus, A. versicolor, Fusarium verticillioides, Microascus manginii, Mucor circinelloides, M. hiemalis, M. plumbeus, M. racemosus, Paecilomyces variotii, Penicillium camemberti, P. commune, P. roqueforti, Scopulariopsis asperula, S. brevicaulis*
Cheese low fat buffalo cottage	*Aspergillus fumigatus*
Cheese Processed	*Aspergillus fumigatus*
Margarine	*Chaetomium globosum, Paecilomyces variotii*
Milk dried non fat	*Scopulariopsis brevicaulis*
Milk	*Aspergillus flavus, Wallemia sebi*

Milk condensed	*Wallemia sebi*
Yoghurt	*Mucor circinelloides* f. *circinelloides* subgroup, *M. hiemalis, M. racemosus*
**Fruit**	
Fruit	*Paecilomyces variotii*
Fruit dried	*Aspergillus flavus, A. niger, Paecilomyces variotii*
Apples	*Alternaria alternata, Aspergillus niger, Fusarium oxysporum, Scopulariopsis brevicaulis, Trichoderma harzianum*
Stored apples	*Acremonium strictum*
Apple peel Lenticel	*Acremonium sclerotigenum*
Apple juice	*Mucor plumbeus, Scopulariopsis brevicaulis*
Bananas	*Alternaria alternata, Acremonium strictum, Fusarium equiseti, F. incarnatum, F. oxysporum, F. proliferatum, F. solani, F. verticillioides, Phoma sorghina*
Banana (Latundan)	*Botryotrichum murorum*
Citrus	*Aspergillus flavus, Fusarium incarnatum, F. verticillioides, F. oxysporum, Mucor racemosus*
Cucurbit fruit	*Fusarium equiseti*
Dates	*Wallemia sebi*
Guavas	*Fusarium solani, Mucor hiemalis*
Grapes	*Aspergillus niger*
Jams	*Wallemia sebi*
Jellies	*Wallemia sebi*
Fruit juices (UHT)	*Fusarium equiseti, F. oxysporum*
Litches	*Aspergillus flavus, Curvularia lunata,*
Mangoes	*Aspergillus niger, Mucor circinelloides*
Mango pickles	*Aspergillus fumigatus*
Melon	*Alternaria alternata, Fusarium incarnatum, F. oxysporum*
Melon (musk)	*Fusarium solani*
Oranges	*Fusarium verticillioides*
Passion fruit	*Fusarium verticillioides*
Pears	*Chaetomium globosum*
Pineapples	*Aspergillus flavus, Fusarium verticillioides*
Pomegranates	*Aspergillus flavus*
Prunes dried	*Wallemia sebi*
Raison	*Aspergillus niger*
Sultanas	*Wallemia sebi*
Tomatoes	*Alternaria alternata, Aspergillus flavus, Chaetomium globosum, Curvularia lunata, Fusarium incarnatum, F. equiseti, F. oxysporum*
**Herbs/Spices/Truffles**	
Bishop's weed (herbal medicine)	*Chaetomium globosum*
Capsicums	*Fusarium equiseti, F. oxysporum, F. solani*
Caraway	*Scopulariopsis brevicaulis*
Cardamom	*Fusarium verticillioides*
Cinnamon	*Chaetomium globosum*
Clover	*Fusarium oxysporum*
Coriander	*Aspergillus flavus, Fusarium incarnatum, F. oxysporum, F. solani, F. verticillioides, Mucor plumbeus*
Chili	*Aspergillus flavus, Chaetomium globosum, Wallemia sebi*
Cumin	*Achaetomium globosum, Chaetomium globosum*
Fennel	*Chaetomium globosum*
Fenugreek	*Fusarium verticillioides*
Ginger	*Chaetomium globosum*
Hops	*Scopulariopsis brevicaulis*
Peppers	*Alternaria alternata, Aspergillus flavus, Chaetomium*

	globosum, Fusarium solani, F. verticillioides, Microascus cinereus, Wallemia sebi
Pepper: black	*Aspergillus flavus, A. tamarii, Chaetomium globosum, Scopulariopsis brevicaulis*
Pepper: red	*Aspergillus flavus, Scopulariopsis brevicaulis*
Pepper: white	*Chaetomium globosum*
Spices	*Aspergillus flavus, A. fumigatus, Dichotomopilus funicola, Curvularia lunata*
Truffles	*Fusarium oxysporum*
**Meat/Eggs/Fish**	
Ham dry cured	*Aspergillus flavus*
Bacon	*Aspergillus flavus, Scopulariopsis brevicaulis*
Biltong	*Alternaria alternata, Fusarium verticillioides, Scopulariopsis brevicaulis*
Eggs	*Scopulariopsis asperula, S. brevicaulis*
Eggs stored	*Aspergillus fumigatus*
Fish meal	*Acremonium sclerotigenum, Scopulariopsis brevicaulis*
Fish dried	*Aspergillus fumigatus*
Fish dried/smoked/cured	*Aspergillus flavus*
Fish dried salt	*Aspergillus flavus, Wallemia sebi*
Ham	*Scopulariopsis brevicaulis*
Meat	*Mucor circinelloides*
Meat (cold/frozen)	*Alternaria alternate*
Meat cured	*Aspergillus fumigatus*
Meat dried cured	*Penicillium commune*
Meat processed	*Aspergillus fumigatus, Mucor racemosus*
Meat products	*Paecilomyces variotii, Wallemia sebi*
Meat smoked	*Aspergillus flavus*
Meat dried	*Aspergillus niger*
Meat salted horse	*Mucor racemosus*
Salmon	*Trichoderma harzianum*
Salami	*Acremonium strictum, Scopulariopsis brevicaulis*
Suet	*Wallemia sebi*
**Nuts/Seeds**	
Almonds	*Aspergillus flavus, A. niger*
Betel	*Aspergillus flavus*
Brazil nuts	*Acremonium recifei, Aspergillus flavus*
Cashew nuts	*Chaetomium brasiliense, C. globosum, Dichotomopilus funicola*
Candle nuts	*Chaetomium globosum*
Coconuts	*Aspergillus flavus*
Copra	*Aspergillus flavus, A. fumigatus, Chaetomium brasiliense, C. globosum, Dichotomopilus funicola*
Hazelnuts	*Alternaria alternata, Aspergillus fumigatus, A. flavus, A. niger, Chaetomium globosum, Curvularia lunata, Fusarium oxysporum, F. verticillioides, Mucor circinelloides, M. hiemalis*
Kemiri nuts	*Aspergillus flavus, Chaetomium globosum*
Kola nuts	*Aspergillus flavus, Fusarium verticillioides*
Tree nuts	*Aspergillus flavus*
Nuts	*Acremonium strictum, Aspergillus flavus, Chaetomium brasiliense, C. globosum, Dichotomopilus funicola, Mucor plumbeus, Paecilomyces variotii, Trichoderma harzianum*
Peanuts	*Alternaria alternata, Aspergillus fumigatus, A. flavus, A. niger, Chaetomium brasiliense, C. globosum, Dichotomopilus funicola, Curvularia lunata, Fusarium chlamydosporum, F. equiseti, F. incarnatum, F. oxysporum, F. solani, F. verticillioides, Lichtheimia*

	corymbifera, Microascus cinereus, Scopulariopsis brevicaulis, Trichoderma harzianum, Wallemia sebi
Pecans	*Alternaria alternata, Aspergillus flavus, Fusarium chlamydosporum, F. oxysporum, F. verticillioides, Microascus trigonosporus, Wallemia sebi*
Pistachios	*Aspergillus flavus, A. niger, A. tamarii*
Walnuts	*Aspergillus fumigatus, A. flavus, A. niger, Chaetomium globosum, Curvularia lunata, Fusarium equiseti, F. oxysporum, Mucor circinelloides*
**Seeds**	
Amaranth	*Aspergillus flavus, Fusarium verticillioides*
Cucumber	*Dichotomopilus funicola, Chaetomium globosum, Botryotrichum murorum*
Legume	*Microascus trigonosporus*
Melon	*Aspergillus fumigatus*
Oilseed	*Aspergillus flavus, A. fumigatus, Fusarium equiseti*
Palm oil	*Fusarium oxysporum*
Pea	*Dichotomopilus funicola, Botryotrichum murorum, Chaetomium globosum, Microascus cirrosus*
Pine	*Scopulariopsis brevicaulis*
Pumpkin	*Botryotrichum murorum, Chaetomium globosum*
Rape	*Alternaria alternata, Aspergillus flavus, Wallemia sebi*
Safflower	*Chaetomium globosum*
Sesame	*Aspergillus flavus, Dichotomopilus funicola, Fusarium solani*
Sunflower	*Aspergillus flavus, A. niger, Fusarium incarnatum, F. solani, F. verticillioides, Scopulariopsis brevicaulis*
**Vegetables**	
Asparagus	*Fusarium verticillioides*
Cabbage	*Fusarium oxysporum*
Carrots	*Mucor hiemalis*
Cassava	*Aspergillus flavus, Fusarium solani, Mucor hiemalis*
Cassava rotting	*Trichoderma harzianum*
Cauliflowers	*Alternaria alternata*
Cucumber	*Alternaria alternata, Fusarium incarnatum, F. oxysporum*
Dates	*Fusarium oxysporum*
Eggplant (aubergine)	*Alternaria alternata, Dichotomopilus funicola,*
Garlic	*Fusarium oxysporum, F. solani, F. verticillioides*
Linseed	*Botryotrichum murorum, Chaetomium globosum, Dichotomopilus funicola*
Maple syrup	*Wallemia sebi*
Oils	*Paecilomyces variotii*
Okra	*Chaetomium globosum*
Olives	*Aspergillus flavus, A. versicolor*
Olive paste	*Lichtheimia corymbifera*
Onions	*Aspergillus niger, Microascus trigonosporus*
Onion dried	*Aspergillus fumigatus*
Peas	*Alternaria alternata, Acremonium strictum, Fusarium oxysporum, F. solani, Trichoderma harzianum*
Peas (chick)	*Aspergillus flavus*
Peas (pigeon)	*Aspergillus flavus, Fusarium incarnatum*
Peas dried	*Wallemia sebi*
Potatoes	*Alternaria alternata, Fusarium incarnatum, F. oxysporum, F. solani, Mucor racemosus*
Radish	*Dichotomopilus funicola*
Squash	*Fusarium solani*
Sugar beets	*Fusarium solani*

Seeds	*Paecilomyces variotii*
Sugarcane	*Chaetomium globosum*
Sweet potatoes	*Fusarium oxysporum, F. solani, Mucor racemosus*
Vegetables (fresh)	*Acremonium strictum, Alternaria alternata, Mucor hiemalis*
Vegetables (tropics)	*Aspergillus fumigatus*
Yams	*Fusarium solani, F. verticillioides, Mucor circinelloides*
Yam chips	*Aspergillus flavus, A. niger*
